# 982 Quality Conversations: Addressing the Problem of Pressure Injuries in Burn ICU

**DOI:** 10.1093/jbcr/iraf019.513

**Published:** 2025-04-01

**Authors:** Carlo Smith

**Affiliations:** Ross Tilley Burn Centre, Sunnybrook Health Sciences Centre

## Abstract

**Introduction:**

In 2023 the results of an annual hospital acquired pressure injury (HAPI) audit showed increased incidence of HAPI in ICUs at our Level 1 Trauma Center. Our ABA Verified Burn Centre was among the units showing increased HAPI. After sharing this data with front line staff we sought to engage them about their perceptions of why there were more HAPIs compared to previous years and how we can move towards HAPI prevention.

**Methods:**

We a quality improvement strategy called Quality Conversations to co-create an HAPI prevention plan with frontline staff. Quality Conversations are staff huddles held at a designed time each week. At these huddles staff were asked to identify root causes of HAPI in burn patients in four domains: The Provider, The Patient, The Organization and The Equipment. This data was collected over several weeks and was enhanced by engaging staff in many formats including: in person; by email; Quality Conversation board posted in the unit allowing staff to add input freely. The most common responses were tabulated and shared with staff. A second phase of Quality Conversation asked staff to explore ways to prevent HAPIs based on the challenges the had identified in the first round. The HAPI prevention strategies derived from staff input were organized into a Safe Turn Checklist specific to our Burn Centre. The list was shared with staff and trailed in patient rooms. The Quality Conversation board was then used to shared monthly HAPI reports with staff including: Summary of incident reports; Percent of device related and non-device related HAPIs; Distribution of HAPI by location; Distribution of HAPI by device.

**Results:**

At total of 79 unique responses were collected and analyzed. Respondents were mainly nurses but also included Personal Support Workers, Administrative Assistants, Physicians, Patient Care Manager, Advanced Practice Nurse, Occupation and Physical Therapists. Results were tabulated on a Pareto Chart and identified 6 root causes of HAPI in Burn patients: Lack of help to support q2h turns; Not removing wet linens; Multiple layers; Heavy patients; Support staff occupied in dressings; high proportion of agency staff who are unfamiliar with unit protocols. Through this exercise and sharing of data, awareness of HAPI and the need to prevent them was enhanced at our Burn Centre.

**Conclusions:**

The Quality Conversation process embodies principles of burn care by providing a forum for staff to engage in quality improvement both individually and as an inter-professional team. Staff voice was used to drive both the root cause analysis and tools for change.

**Applicability of Research to Practice:**

Our use of Quality Conversations demonstrates the power of shared governance and staff engagement to drive increased awareness and dissemination of best practices at the bedside. We believe that burn teams are an invaluable resource. As such, leaders must show staff caring for burn patients that they are valued by hearing and implementing their ideas whenever possible.

**Funding for the Study:**

We did not receive funding for this project.

